# Favorable efficacy of adalimumab treatment in experimental acute pancreatitis model

**DOI:** 10.55730/1300-0144.5528

**Published:** 2022-09-12

**Authors:** Başak ÇAKIR GÜNEY, Alpaslan TANOĞLU, Murat YENİÇERİ, Zafer ÇIRAK, Ayşe Gökçen SADE, Yeşim ÖNAL TAŞTAN, Zeliha SERİNDAĞ, Mustafa KAPLAN

**Affiliations:** 1 Department of Internal Medicine, Sultan 2.Abdülhamid Han Training and Research Hospital, University of Health Sciences, İstanbul, Turkey; 2 Department of Gastroenterology, Sancaktepe Şehit Prof. Dr. İlhan Varank Training and Research Hospital, University of Health Sciences, İstanbul, Turkey; 3 Department of Pathology, Sultan 2.Abdülhamid Han Training and Research Hospital, University of Health Sciences, İstanbul, Turkey

**Keywords:** Acute pancreatitis, Adalimumab, inflammation, apoptosis

## Abstract

**Background/aim:**

Acute pancreatitis is a clinical picture with a wide range of symptoms from mild inflammation to multiorgan failure and death. The aim of this study is to investigate the effects of Adalimumab (ADA) on inflammation and apoptosis in a cerulein-induced acute pancreatitis model in rats.

**Material and methods:**

Experimental cerulein-induced acute pancreatitis model was created by applying 4 intraperitoneal cerulein injections at 1-h intervals. A total of 40 rats, 8 in each group, were randomly distributed into five groups. In the groups that ADA treatment was given, two different doses of ADA were administered 5 mg/kg and 20 mg/kg as low and high doses, respectively. The rats were sacrificed 12 h after the last intraperitoneal administration of ADA. Blood samples were obtained from each rat for amylase, IL-6, and IL-1β measurements. Hematoxylin and Eosin (H&E) stains were used to undertake the histopathological analysis of the pancreas. The terminal deoxynucleotidyl transferase-mediated nick-end-labeling (TUNEL) method was used to evaluate apoptosis.

**Results:**

Plasma amylase, IL-6, and IL-1β levels were significantly elevated in acute pancreatitis groups (p < 0.05). It was determined that both low (5 mg/kg) and high doses (20 mg/kg) of ADA ameliorated the parameters (plasma amylase, IL-6, and IL-1β) (p < 0.05). Although significant improvements were detected in the Schoenberg scoring system and the apoptotic index from the TUNEL method after high-dose ADA treatment, no significant amelioration was observed in the histopathological examinations in the low-dose ADA group.

**Conclusions:**

It has been determined that the administration of high-dose ADA effectively alleviated the symptoms of acute pancreatitis and reduced the level of apoptosis. In line with the findings of our study, we have predicted that high-dose (20 mg/kg) ADA can be used as an effective and safe drug in the treatment of patients with acute pancreatitis.

## 1. Introduction

Acute pancreatitis (AP) constitutes an important part of the indications for emergency hospitalization in gastroenterology and internal medicine clinics all over the world [1]. AP is an acute and severe inflammation of the pancreas that develops due to various factors [2]. Mild abdominal pain is among the possible symptoms. However, it is a process with an increased inflammatory response that often manifests itself with acute, severe abdominal pain and accompanying elevated pancreatic enzymes (amylase, lipase) [3]. Despite recent advances in gastroenterology, AP is still strongly linked to morbidity, mortality, and utilization of healthcare resources [4]. AP has a 1.5% mortality rate in mild cases. This rate rises to 17% in severe cases [5,6]. Bile duct diseases (like gallstones) and alcohol use constitute most of the etiology of AP. In the pathogenesis of AP, there is a complex series of immunological responses that affect the course of the disease. Regardless of the cause that initiates the response, this locally initiated inflammatory response may become widespread and cause multi-organ damage [7]. Autodigestion caused by digestive enzymes released from pancreatic acinar cells is the primary trigger of local inflammation. This autodigestion spreads to the retroperitoneal sites over time, creating widespread necrotic areas. These necrotic tissues trigger infection over time, causing sepsis and multiple organ damage [8].

Cerulein is an oligopeptide consisting of 10 amino acids. It was first obtained from the skins of Australian green frogs. It is very similar in structure to cholecystokinin. Therefore, it triggers pancreatitis in experimental animal models with a mechanism similar to pancreatic autodigestion [9]. Cerulein, in addition to triggering inflammation in acinar cells, causes dysregulation in pancreatic digestive enzyme production. It causes acinar cell death and pancreatic edema. Cerulein also causes the activation of NADPH oxidase, a source of reactive oxygen species that contribute to inflammation, and Janus kinase, which is another trigger of inflammation [10]. In this study, intraperitoneal cerulein was used to create pancreatitis in rat models.

It has been known for many years that proinflammatory cytokines are involved in the physiopathology of AP. TNF (tumor necrosis factor)-α, ICAM-1, interleukin (IL)-1, IL-6, IL-8, platelet-activating factor (PAF), and MCP (monocyte chemoattractant protein)-1 are pro-inflammatory mediators that play a role in AP [7]. TNF- is a cytokine produced by macrophages and lymphocytes. In previous studies, TNF-α was found to be increased in patients with pancreatitis and in animal models [11–13]. Recombinant human IgG1 monoclonal antibody Adalimumab (ADA) specifically targets human TNF-α. ADA inhibits both superficial cell receptors of TNF-α, type-I (p55), and type-II (p75). Because of this effect, ADA is a treatment that has been approved for psoriasis, psoriatic arthritis, and rheumatoid arthritis for years. In recent studies, it has been shown that TNF-α inhibition in experimental animal models is beneficial in AP [14,15]. In a study conducted on rats in 2010, the effects of ADA in (Na)-taurocholate-induced pancreatitis were evaluated. The beneficial effects of ADA at a dose of 50mg/kg on pancreatitis have been demonstrated histopathologically and biochemically [16].

In this study, it is aimed to examine the effects of ADA, which we predict can be used as a new therapeutic agent in acute pancreatitis that still has no curative treatment, on inflammation and apoptosis in an experimental acute pancreatitis model.

## 2. Materials and methods

### 2.1. Study design

In this study, 40 Spraque-Dawley rats (250–350 g) were used. These rats were kept in cages at approximately 24 °C in 12-h day-night cycle. The rats were allowed free water and food, and their food intake was stopped 12 h before injections. The study was carried out within the framework of national guidelines for the care and use of laboratory animals. After obtaining approval from the University of Health Sciences Hamidiye Experimental Animals Ethics Committee, the experimental procedures were performed in the University of Health Sciences Hamidiye Medical Faculty Experimental Animals Laboratory. Acute pancreatitis was induced by intraperitoneal injection of cerulein (Sigma, St. Louis, MO, USA) administered 4 times at 1-h intervals.

A total of 40 rats, 8 rats in each group, were randomly distributed into five groups.

Group 1: Sham group (1mL of saline was administered to rats 4 times intraperitoneal injection at 1-h intervals, rats were sacrificed 24 h after the last injection).
Group 2: Acute pancreatitis (AP) group (intraperitoneal injection of 40 μg/kg cerulein in saline 4 times at 1-h intervals. Rats were sacrificed 24 h after the last injection).
Group 3: AP + low-dose ADA group (intraperitoneal injection of 40 μg/kg cerulein in saline 4 times at 1-h intervals, then a single dose intraperitoneal injection of 5 mg/kg ADA 12 h after the last cerulein injection. The rats in this group were sacrificed 12 h after the last ADA injection).
Group 4: AP + high-dose ADA group (intraperitoneal injection of 40 μg/kg cerulein in saline 4 times at 1-h intervals, then a single dose intraperitoneal injection of 20 mg/kg ADA 12 h after the last cerulein injection. The rats in this group were sacrificed 12 h after the last ADA injection).
Group 5: Placebo group (intraperitoneal injection of 40 μg/kg cerulein in saline 4 times at 1-h intervals, then intraperitoneal injection of 1 cc saline as placebo 12 h after the last cerulein injection. The rats in this group were sacrificed 12 h after saline administration).

### 2.2. Randomized controlled trials

Randomization is a process used to ensure that each experimental unit has an equal probability of receiving a particular treatment. An experiment with 5 treatment groups (1: Sham, 2: Pancreatitis 3: Adalimumab 5 mg/kg 4: Adalimumab 10 mg/kg, and 5: Placebo) with 8 animals per group. The function = Rand () in Excel can be used to generate a column of random numbers in column A. Column B would contain eight 1’s, eight 2’s, eight 3’s, eight 4’s and eight 5’s for each of the treatment groups and column C would contain unique identification numbers for each of the 40 animals. Sorting columns A and B by the order of column A will randomize the order of column B and each animal of column C will be allocated into treatment 1, 2, 3, 4, or 5 at random.

The number of samples to be taken for the study was determined by GPOWER 3.1.9.4. Statistically, a total of 40 rat plans were calculated with 0.85 power 0.05 error 0.065 effect size files. Amylase (U/l) values for 5 groups for effect size.

**Figure 1:**
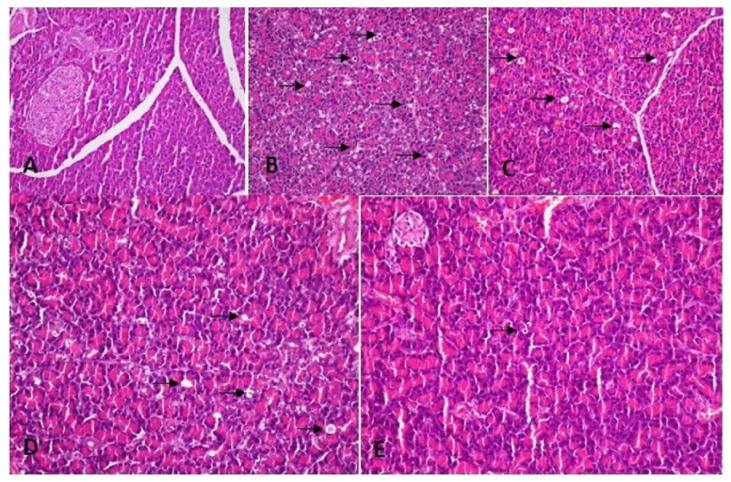
Hematoxylin and eosin staining of pancreatic tissue samples. A. Control group with normal pancreatic tissue, 20× magnification. B. Acute pancreatitis group, intralobular polymorphonuclear cell infiltration, vacuolation, necrosis and edema are seen, 20x magnification. C. Sp treated placebo group, vacuolization, edema, and inflammation are still existing, 20× magnification. D. Adalimumab treatment group, reduced polymorphonuclear cells, vacuolation, and edema with low dose, 20× magnification. E. Adalimumab treatment group with high dose, 20× magnification. (Polymorphonuclear cell infiltration, vacuolation, necrosis, and edema are marked with black arrows)

**Figure 2:**
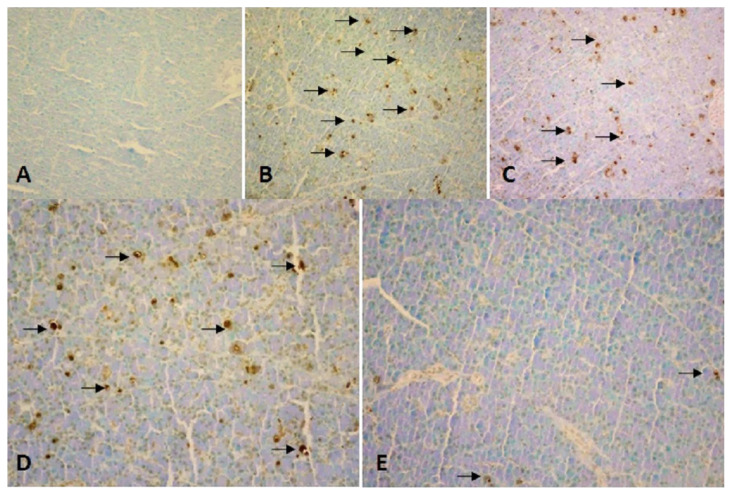
TUNEL technique shows the apoptotic status of study groups. A. Control group. No apoptotic cell is seen. B. Acute pancreatitis group. Increased apoptotic cells are seen. Apoptotic index is 4.7%. C. Sp treated placebo group, similar features with acute pancreatitis group. Apoptotic index is 5.3%. D. Adalimumab treatment group. Decreased apoptotic cells are seen with a low dose. Apoptotic index is 3.55% E. Adalimumab treatment group. Apoptotic index is 2.08% (TUNEL staining, ×20 magnification. Apoptotic cells are marked with black arrows).

Blood samples were collected by intracardiac route from the rats. The pancreas tissues were then quickly removed, and the rats were euthanized with high-dose anesthesia. Then blood samples of animals were centrifuged for 10 min at 3500 rpm and the plasma samples were stored at −80 °C until analyses. Amylase, IL-1β, and IL-6 levels were measured in plasma samples. Commercial enzyme-linked immunosorbent assay (ELISA) kits were used to determine the plasma amylase (Scientific Research Special Hangzhou Eastbiopharm Co. Ltd. Hangzhou), IL-6 (Scientific Research Special Hangzhou Eastbiopharm Co. Ltd. Hangzhou), and IL-1β (AssayPro, USA) levels. Analyses were carried out according to the instructions and guidelines of manufacturers. Pancreatic tissue samples of rats were fixed in 10% formaldehyde. Preparations were stained with hematoxylin and eosin (H&E). They were cut into 5 μm-thick sections stained with hematoxylin and eosin. Samples were numbered from one to forty. The pathologist was not informed about the treatments given for blind evaluation. Edema, acinar necrosis, bleeding, inflammation, and the presence of perivascular infiltration were examined and evaluated under a photomicroscope (Olympus BX 51; Tokyo, Japan) according to the Schoenberg grading system. It was graded between 0 and 4 according to the Schoenberg grading system (Figure 1) [17]. Terminal deoxynucleotidyl transferase-mediated nick-end-labeling (TUNEL) method was used to examine apoptotic cells and for examination, a commercial kit (ApopTag Plus Peroxidase, In Situ Apoptosis Detection Kit, Chemicon Int., Temecula, CA) was used. The nuclei are expected to be blue in normal cells from pancreatic samples prepared with the TUNEL technique. The nuclei of apoptotic cells appear brown. The percentages of apoptotic cells were calculated according to the number of cells in the area where the acinar cells were most heavily stained (Figure 2).

### 2.3. Statistical analysis

Statistical evaluation was performed with the Statistical Package for Social Sciences (SPSS 20.0 for windows). The conformity of the data to the normal distribution was checked with the Shapiro-Wilk test, and the homogeneity of the variances was checked with the Levene test. The results were expressed as Mean ± SD or Median (min-max). Comparisons of means between groups were evaluated with one-way analysis of variance (ANOVA) (Post Hoc: Bonferonni) for normally distributed data and the Kruskal-Wallis test (Post Hoc: Mann-Whitney U test with Bonferroni correction) for nonparametric data. p < 0.05 and p < 0.005 (Bonferroni correction) were considered as statistically significant and two-sided tests were applied.

## 3. Results

### 3.1. Biochemical examination

Plasma amylase, IL-6, and IL-1β levels were examined according to the instructions of the ELISA kit manufacturer (AssayPro, USA, Bioscience, Austria and Scientific Research Private Hangzhou Eastbiopharm Co. Ltd. Hangzhou, repeatedly). Results are shown in Table 1. Plasma amylase, IL-6, and IL-1β levels in the groups with acute pancreatitis were significantly increased when compared to the sham group (p < 0.01). Pancreatitis control (Group 2) and pancreatitis placebo (Group 5) groups did not differ statistically. On the other hand, plasma amylase level, IL-6, and IL-1β levels were significantly decreased in the ADA-treated groups (Groups 3 and 4) compared to the control group (p < 0.05). When the groups receiving low-dose ADA (Group 3) and high-dose ADA (Group 4) were compared, no significant difference was found between the two groups (Table 2).

### 3.2. Histopathological evaluation

For histopathological evaluation, pancreatic tissue samples were fixed using 10% buffered formaldehyde and embedded in paraffin. Tissue sections were stained with hematoxylin and eosin (H&E) and taken for examination under a photomicroscope (Olympus BX 51; Tokyo, Japan). Damages in tissues were classified by applying the Schoenberg grading system.

Significant edema, inflammation, vacuolization, and fat necrosis were detected in all cerulein injected groups compared to the sham group (Table 3). There was no significant improvement in pancreatitis control and pancreatitis placebo groups in the low-dose ADA group (Group 3). However, a significant improvement was found in the group receiving high-dose ADA (Group 4) compared to all groups with pancreatitis (p < 0.01) (Table 4). A commercial kit (ApopTag Plus Peroxidase, In Situ Apoptosis Detection Kit, Chemicon Int., Temecula, CA) was used to evaluate apoptosis in pancreatic tissues with TUNEL method. Apoptotic indices were found to be 4.7% and 5.3% in the pancreatitis control group (Group 2) and the placebo group (Group 5), respectively. The apoptosis index was 3.55% in the group that received low-dose ADA (Group 2) with a minimal decrease (Table 5). However, in the group receiving high-dose ADA (Group 5), the apoptosis index was 1.84% which was a marker of significant improvement.

## 4. Discussion

Proinflammatory cytokines are the most prominent factors in the pathogenesis of local and systemic effects of acute pancreatitis. There is a serious correlation between the presence of an increased proinflammatory cytokine response in the early phase of the disease, the inadequacy of the corresponding antiinflammatory response, and the severity of the disease [18]. Through the activation and adherence of inflammatory cells, the release of cytokines, nitric oxide (NO), and inflammatory mediators, TNF-α is known to mediate tissue injury. Neutrophils activated by TNF-α release free radicals, causing diffuse tissue edema, lipid peroxidation, and ultimately cell death [19,20]. The search for a new treatment that can change the course of acute pancreatitis is still ongoing. It has been predicted that TNF-alpha inhibition may affect the course of the disease by suppressing local and systemic inflammation. Some studies have also been done on experimental animal models before [12,13]. Adalimumab is a TNF-α inhibitor used in clinical practice in the treatment of many diseases such as psoriasis, psoriatic arthritis, rheumatoid arthritis, ankylosing spondylitis, inflammatory bowel diseases (Crohn’s disease and ulcerative colitis), and juvenile idiopathic arthritis [21].

Yilmaz et al. showed the beneficial effects of ADA at a dose of 50 mg/kg on sodium taurocholate-induced pancreatitis in rats [16]. In this study, it was observed both biochemical and histopathological improvements in pancreatitis at a dose of 20 mg/kg. Grewalet et al. stated that TNF-α levels increased in acute pancreatitis and this cytokine may play a role in the pathogenesis of the disease. Researchers demonstrated that a statistically significant improvement in biochemical parameters was achieved by using polyclonal blockade of TNF-α [12]. Hughes et al. showed that anti-TNF antibodies are beneficial in curing acute pancreatitis and its related complications in rats [13]. Norman et al., using a soluble antagonist of the type I TNF receptor (TNFbp), reported that it reduced the course and mortality of experimentally induced pancreatitis [22]. In this study, it was found that inflammation was suppressed better, and apoptosis was lower in the high-dose ADA treatment group. It is obvious that ADA, which is quite safe in terms of its side effect profile, will be more effective in the treatment of acute pancreatitis at higher doses. However, the results of the study cannot be directly applied to humans without human studies.

## 5. Conclusion

Our study shows that ADA is a new alternative in the treatment of AP with suitable doses. Our study predicts that ADA, which is used in daily practice in many diseases, can be used in the treatment of pancreatitis. If AP is not treated urgently, its acute and chronic effects can have life-threatening consequences. For this reason, it is obvious that there is a need for new treatment alternatives for pancreatitis which has limited treatment options. ADA may be an effective and correct treatment option in patients with acute pancreatitis. However, more human studies are needed to evaluate the effectiveness of ADA in people with acute pancreatitis. Studies on this subject in new and larger series are required.

## Figures and Tables

**Table 1:** The biochemical parameters of the study groups.

Groups	Amylase (U/L)	Il-6 (pg/mL)	IL-1β (pg/mL)
Group 1	77.28 (63–98)	28.76 (22–52)	33.5 (20–58)
Group 2	477.13 (401–611)	605.08 (579–670)	145.62 (128–171)
Group 3	279.65 (221–360)	515.45 (431–590)	114.88 (81–130)
Group 4	225.48 (215–270)	404.5 (301–528)	77.28 (63–93)
Group 5	479.32 (421–610)	774.96 (725–866)	152.53 (101–170)
**p**	<0.0001^KW^	<0.0001^KW^	<0.0001^KW^

Data are given as median (min-max).

KW Kruskal Wallis

**Table 2:** Statistical parameters of the study groups.

Groups	Amylase (U/L)	IL-6 (pg/mL)	IL-1β (pg/mL)
Groups 1–2	p < 0.01	p < 0.01	p < 0.01
Groups 1–3	p < 0.01	p < 0.01	p < 0.01
Groups 1–4	p < 0.01	p < 0.01	p < 0.01
Groups 1–5	p < 0.01	p < 0.01	p < 0.01
Groups 2–3	p < 0.01	p < 0.05	p < 0.05
Groups 2–4	p < 0.01	p < 0.01	p < 0.05
Groups 2–5	NS	NS	NS
Groups 3–4	NS	NS	NS
Groups 3–5	p < 0.01	p < 0.05	p < 0.05
Groups 4–5	p < 0.01	p < 0.01	p < 0.01

Abbreviation: NS: Not significant. Mann-Whitney U test with Bonferroni correction multiple comparison. The mean difference is significant at the p < 0.05 level and p < 0.005 is considered statistically significant after Bonferroni’s correction.

**Table 3:** Schoenberg scores of the study groups.

Groups	Edema	Inflammation	Vacuolation	Necrosis	Score
Group 1 (Sham)	0	0	0	0	0
Group 2 (Pancreatitis)	3.25 ± 0.5	3.00	2.75 ± 0.5	3 ± 0.81	12 ± 0.81
Group 3 (Adalimumab 5 mg/kg)	3.28 ± 0.48	2.57 ± 0.53	2.14 ± 0.69	2.85 ± 0.69	10.85 ± 1.67
Group 4 (Adalimumab 10 mg/kg)	2.28 ± 0.48	1.57 ± 0.78	1 ± 0.57	1.14 ± 0.37	6 ± 1.15
Group 5 (Placebo)	3.75 ± 0.5	3 ± 0.81	2.75 ± 0.5	2.75 ± 0.5	12.25 ± 1.70
**P**	0.005^A^	<0.00001^A^	0.007^A^	0.0009^A^	<0.00001^A^

A One-way analysis of variance (ANOVA)

**Table 4:** Statistical Schoenberg scores of the study groups.

Groups	Schoenberg scores*
Groups 1–2	p < 0.001
Groups 1–3	p < 0.001
Groups 1–4	p < 0.001
Groups 1–5	p < 0.001
Groups 2–3	NS
Groups 2–4	p < 0.001
Groups 2–5	NS
Groups 3–4	p < 0.01
Groups 3–5	NS
Groups 4–5	p < 0.05

Abbreviation: NS: Not significant, Bonferroni, multiple comparison.

* The mean difference is significant at the 0.05 level considered statistically significant.

**Table 5:** Apoptotic scores.

Groups	Apoptotic scores
Group 2	4.70%
Group 3	3.55%
Group 4	1.84%
Group 5	5.30%
